# Determination of time of death by blinded post-mortem interrogation of cardiac implantable electrical devices

**DOI:** 10.1038/s41598-022-12390-3

**Published:** 2022-05-17

**Authors:** Korbinian Lackermair, Florian Fischer, Johannes Manhart, Eva Scheurer, Matthias Graw, Diana Boy, Claudia Lenz, Bonnie Hartrampf, Antonia Kellnar, Lauren Sams, Heidi Estner, Stephanie Fichtner

**Affiliations:** 1 Department of Medicine I, University Hospital Munich, Ludwig Maximilian University, Marchioninistr. 15, 81377 Munich, Germany; 2 Institute of Legal Medicine, Ludwig-Maximilian University, Munich, Germany; 3 Institute of Legal Medicine, Rostock University Medical Center, Rostock, Germany; 4 Department of Biomedical Engineering, Institute of Forensic Medicine, University of Basel, Basel, Switzerland; 5 Institute of Forensic Medicine, Health Department Basel-Stadt, Basel, Switzerland

**Keywords:** Cardiac device therapy, Pathology

## Abstract

Postmortal interrogation of cardiac implantable electrical devices (CIED) may contribute to the determination of time of death in forensic medicine. Recent studies aimed to improve estimation of time of death by combining findings from autopsy, CIED interrogation and patients´ medical history. CIED from deceased undergoing forensic autopsy were included, if time of death remained unclear after forensic assessment. CIED explanted from deceased with known time of death were analysed as a control cohort. CIED were sent to our device interrogation lab and underwent analysis blinded for autopsy findings, medical history and police reports. The accuracy of time of death determination and the accuracy of time of death in the control cohort served as primary outcome. A total of 87 CIED were analysed. The determination of time of death was possible in 54 CIED (62%, CI 52–72%). The accuracy of the estimated time of death was 92.3% in the control cohort. Certain CIED type and manufacturers were associated with more successful determination. Blinded postmortal analysis enables a valid determination of the time of death in the majority of CIED. Analysis of explanted CIED in a cardiological core lab is feasible and should be implemented in forensic medicine.

## Introduction

Cardiac implantable electrical devices (CIED) represent an important treatment of cardiac arrhythmia. Pacemakers (PM) provide sufficient therapy in patients suffering from bradycardia whereas implantable cardioverter defibrillators (ICD) play an important role in prevention of sudden cardiac death (SCD) caused by ventricular tachycardia or ventricular fibrillation^1–4^. Contemporary CIED systems are complex microcomputers, which provide tailored therapies due to individualized programming. Besides automatic surveillance of lead function, modern CIED also enable the analysis of diagnostic data like heart rate trends, percentages of pacing, storage of arrhythmia or additional information like estimation of pulmonary congestion, patient´s activity or abnormalities in repolarization^5,6^. Individualised programming of CIED systems is possible and stored information can be analysed percutaneously using manufacturer specific programmers.

Previous reports have shown the high informative value of postmortal analysis of CIED in addition to findings from forensic autopsy^7–14^. It has been shown, that postmortal interrogation may clarify time and, in some cases, even cause of death^9,10,15^.

The 2017 guidelines of the Association for European Cardiovascular Pathology recommended CIED removal and interrogation at autopsy in deceased with a potential sudden cardiac death^16^. Nevertheless, postmortal analysis of CIED is not routinely performed in forensic autopsy of patients with unknown time of death, even if this remains unclear after autopsy. CIED analysis in the context of forensic autopsy has three major limitations: First, technical analysis of CIED is complicated by the need for a manufacturer specific proprietary programmer. Second, valid postmortal analysis of CIED is complex and exceeds personal qualification requirements compared to routine CIED interrogation. And finally, prior studies performed analyses, which combined information from CIED analysis, autopsy and medical history. Thus, no data are available about the information generated from the CIED analysis alone.

The purpose of this prospective multicentre postmortal CIED trial was to overcome these limitations. The primary questions were:

- Does postmortal CIED analysis blinded for any information about patient´s history and autopsy findings provide sufficient information about time of death?
- Is performance of CIED analysis in a device interrogation lab (in part relevantly distant from the site of autopsy) feasible?

## Methods

### Study design

The postmortal CIED trial was designed as a prospective multicentre study and conducted in accordance with the declaration of Helsinki after approvement by the ethics committee of the University of Munich (accession number 17-583) as well as by the local ethics committees of Rostock (accession number A2019-0102), Bonn (accession number 246/19), Freiburg (accession number 302/19), and Basel (Switzerland, accession number 2019-01,499). As CIED would in every case have been explanted and analysis did not change that the CIED were destroyed after forensic examination was completed, our analysis did not burden the deceased additionally. Therefore, consent was not required from next of kin or other legal representatives according to all ethics committees. Recruitment started in January 2019 at our local department of legal medicine. The study protocol planned the initiation of a total of 17 legal medicine departments in Germany, Austria and Switzerland to include a total of 200 CIED. As initiation was significantly impeded by regulatory restrictions caused by the COVID-19 pandemic, the trial was stopped in December 2020.

### Study subjects

Inclusion criteria were the presence of a CIED in deceased undergoing forensic autopsy in addition to time (and cause) of death remaining unclear after autopsy. The forensic departments were advised to explant CIED during autopsy by using the appropriate screw driver to avoid cutting of CIED leads. The CIED was sent by mail to the core lab at our cardiology department (Department of Medicine I, University Hospital Munich, Ludwig Maximilian University, Munich) without any information besides the time of autopsy.

A control cohort of CIED explanted from subjects with known time of death undergoing autopsy at our local legal medicine department (Institute of Legal Medicine, Ludwig-Maximilian University, Munich) during the study period was included to assess the accuracy of the determination of time of death.

### CIED interrogation

All interrogations were performed with device specific programmers by the same electrophysiologist and specialist for CIED therapy at the Department of Medicine I, University Hospital Munich, Ludwig Maximilian University, Munich.

**Figure 1:**
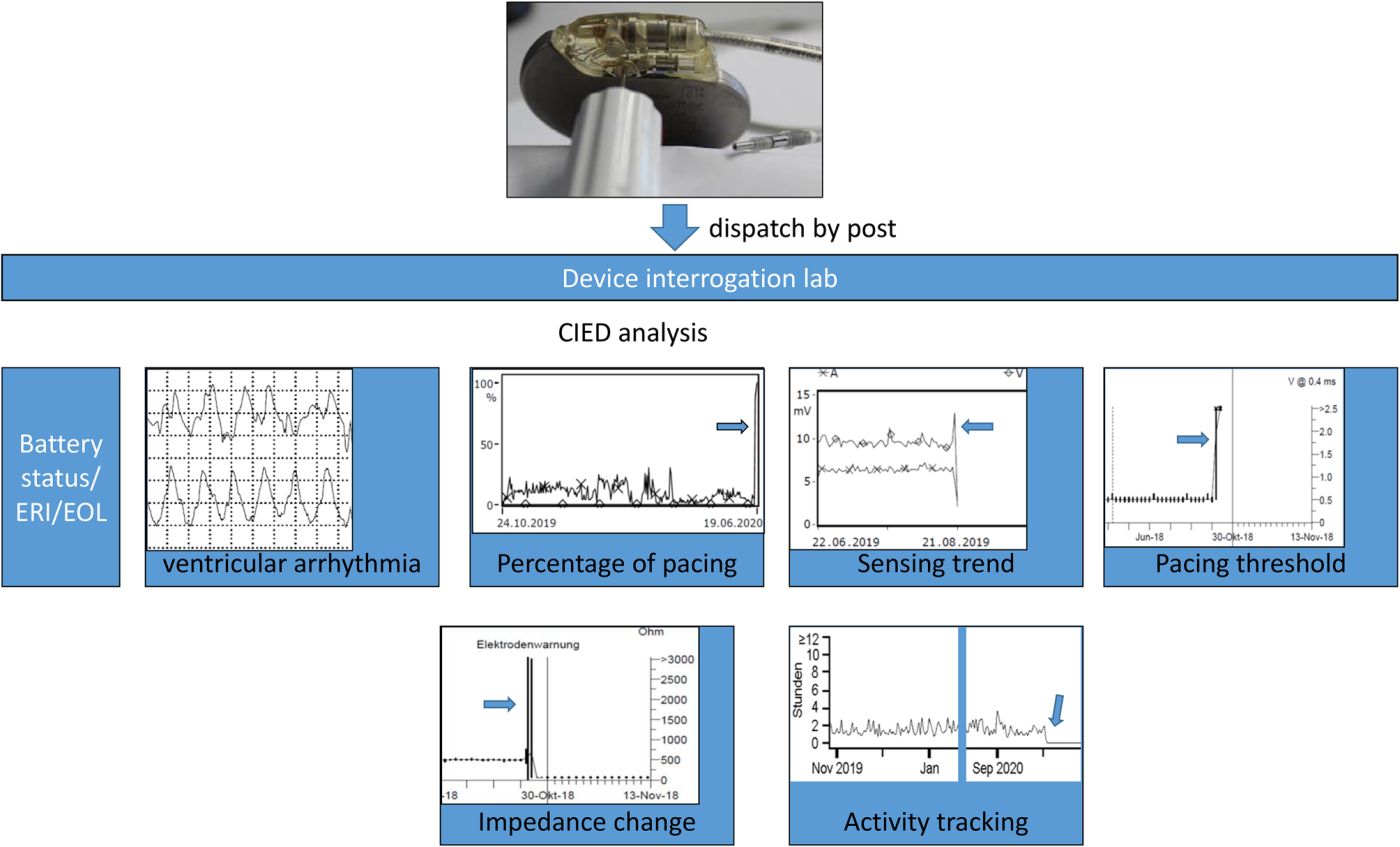
Device interrogation. Firstly, leads were removed using a specific screw driver to avoid lead cutting noise overwriting the internal device memory. Thereafter the device was sent to the device interrogation core lab and was analysed blinded to autopsy results.

The interrogation’s primary objective was to determine the time of death or any signs for an arrhythmic cause of death. The approach for the determination of time of death was described earlier^10^. Briefly, determination of time of death was made by documented, most probably lethal arrhythmia, analysis of changes in lead parameters, percentage of pacing or additional diagnostic data (Fig. 1).

### Statistical analysis

Categorical data were expressed as absolute and relative frequencies. Continuous data were expressed as mean and interquartile range (IQR). Group comparisons between categorical variables were performed with the Pearson χ2 test for rates. Subgroups with a very small number of cases were excluded from comparisons by statistical tests. All statistical analyses were performed by SPSS 26 (IBM, New York, USA).

### Funding

This investigator initiated trial was funded by unrestricted grants by Abbott, Boston Scientific and BIOTRONIK, which had no role in the design of the trial, analysis of the data, or the drafting and submission of the manuscript.

## Results

**Figure 2:**
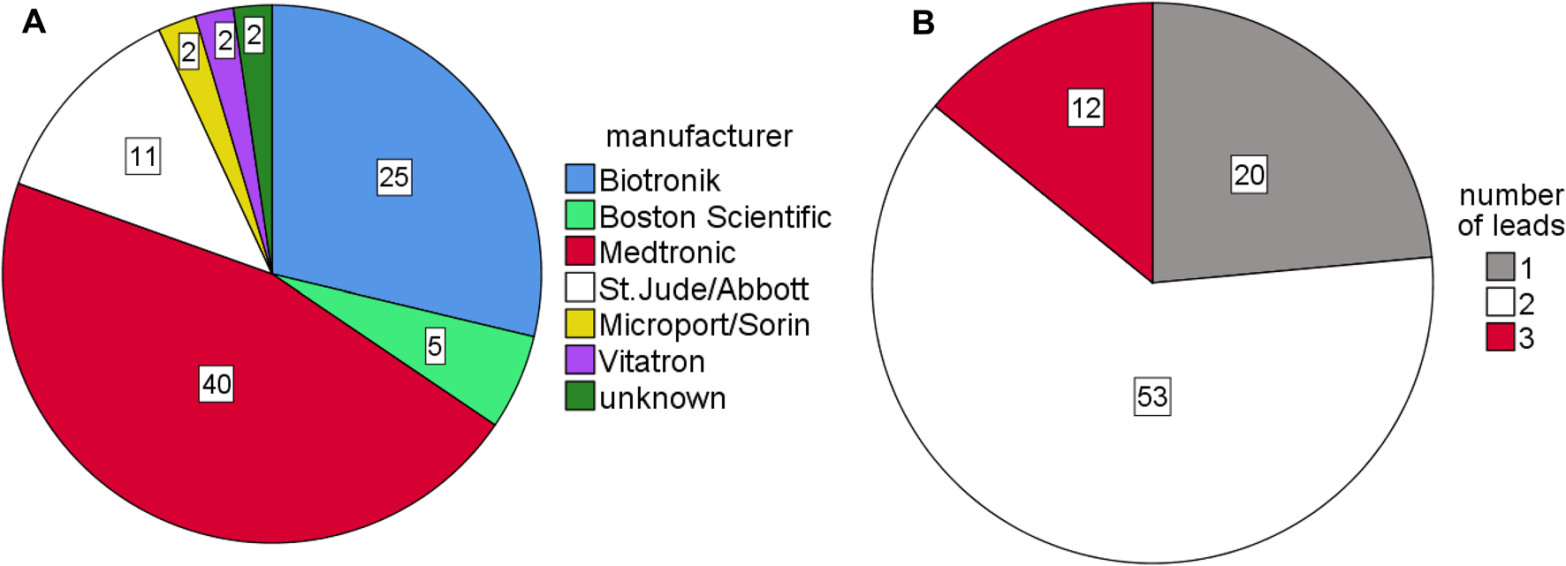
Pie charts illustrating the composition of the CIED cohort. (**A**): CIED manufacturers are depicted. In 2 CIED total battery depletion disabled interrogation. CIED type and manufacturer were not recorded. (**B**): CIED analysed were predominantly dual chamber devices. Devices for cardiac resynchronisation therapy (with 3 leads) played a minor role.

A total of 87 CIED underwent analysis. Of these, 21 CIED (24%) were explanted from deceased with known time of death and served as control cohort. The group consisted of 22 ICD und 65 Pacemakers. The manufacturer were predominantly Medtronic (n = 40; 46%) and BIOTRONIK (n = 25; 29%; Fig. 2A). CIED were primarily single or dual chamber devices (n = 73; 84%) whereas cardiac resynchronisation devices (CRT) played a minor role (n = 12, 14%, Fig. 2B). The battery status was regular in 68 CIED (78%). Battery backup (ERI) was active in 15 CIED, which leads to preserved pacing function but limited diagnostic features. One PM was found in emergency pacing program due to imminent total battery depletion and could not be analysed. This device was sent to the manufacturer for further examination. The manufacturer found regular battery depletion after an operating time of 13 years but no further hardware concern. Three PM presented with total battery depletion and without ongoing pacing. Shock therapies were found deactivated after death in two ICD devices (9%). The characteristics of the examined CIED and their functional state are depicted in supplemental table 1.

The determination of time of death was possible in 54 CIED (62%; CI 52-72%). No difference was seen with respect to estimation rates between the control cohort (with known time of death) and the study cohort (62 vs. 62%). In the control cohort (total estimation rate 62%) estimation was in accordance with the documented time of death in 12 of 13 CIED (92%).

**Figure 3:**
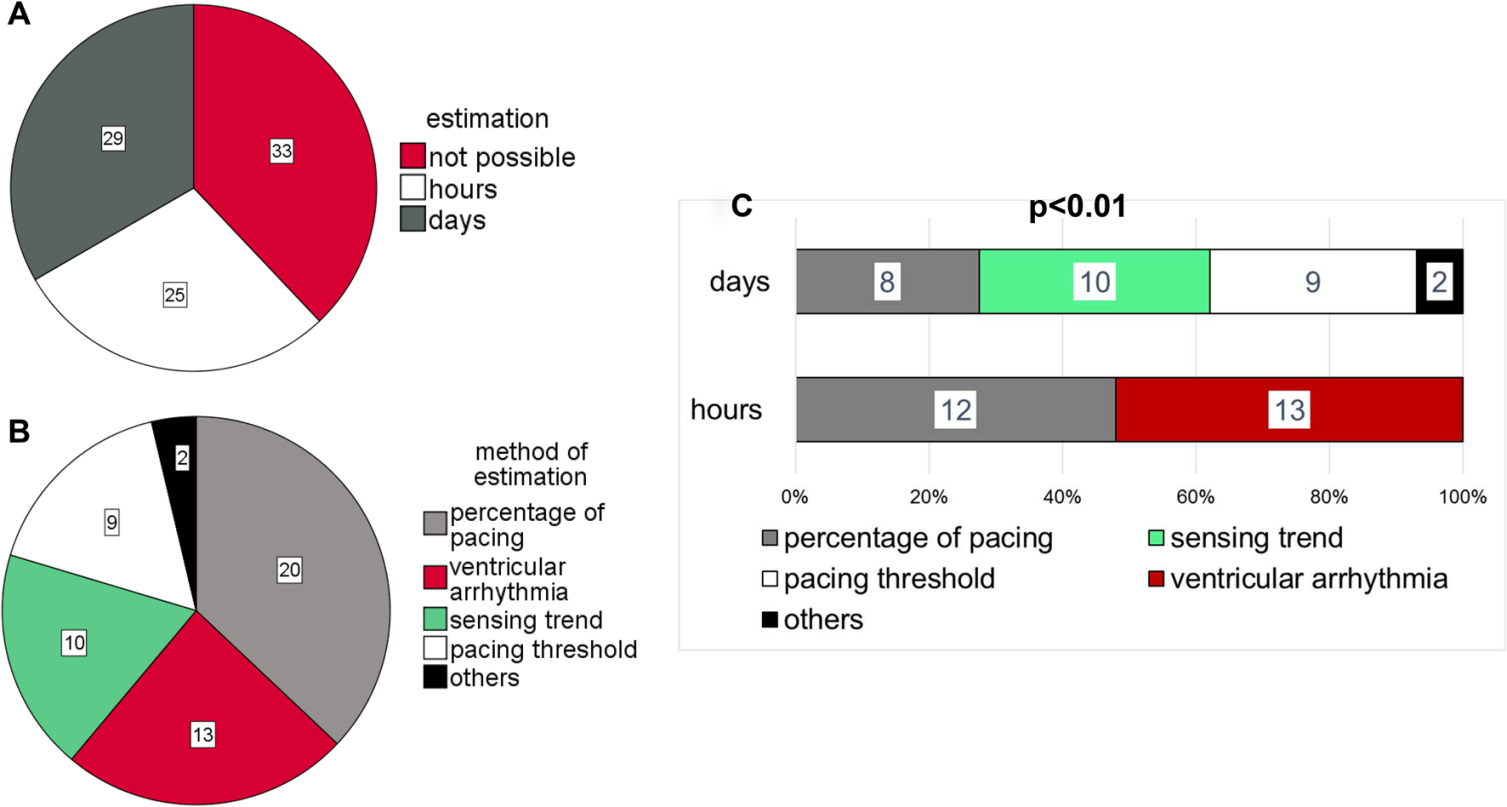
(**A**): Pie chart depicting the success of the determination of time of death (not possible, exact within a day, exact within an hour). (**B**): Primary method of estimation is depicted. (**C**): A significant difference in the primary method of estimation is visualized between CIED with an estimation of time of death within an exact hour and within a day (*p* < 0.01).

The estimation of time of death was exact within a day in 29 CIED (33%) and within an hour in 25 CIED (29%, Fig. 3A). The primary method of this estimation for the total cohort and differences between the methods used for the estimation the hour or day of death between deceased with an ICD or PM are depicted in Fig. 3B,C and supplemental Fig. 1.

**Figure 4:**
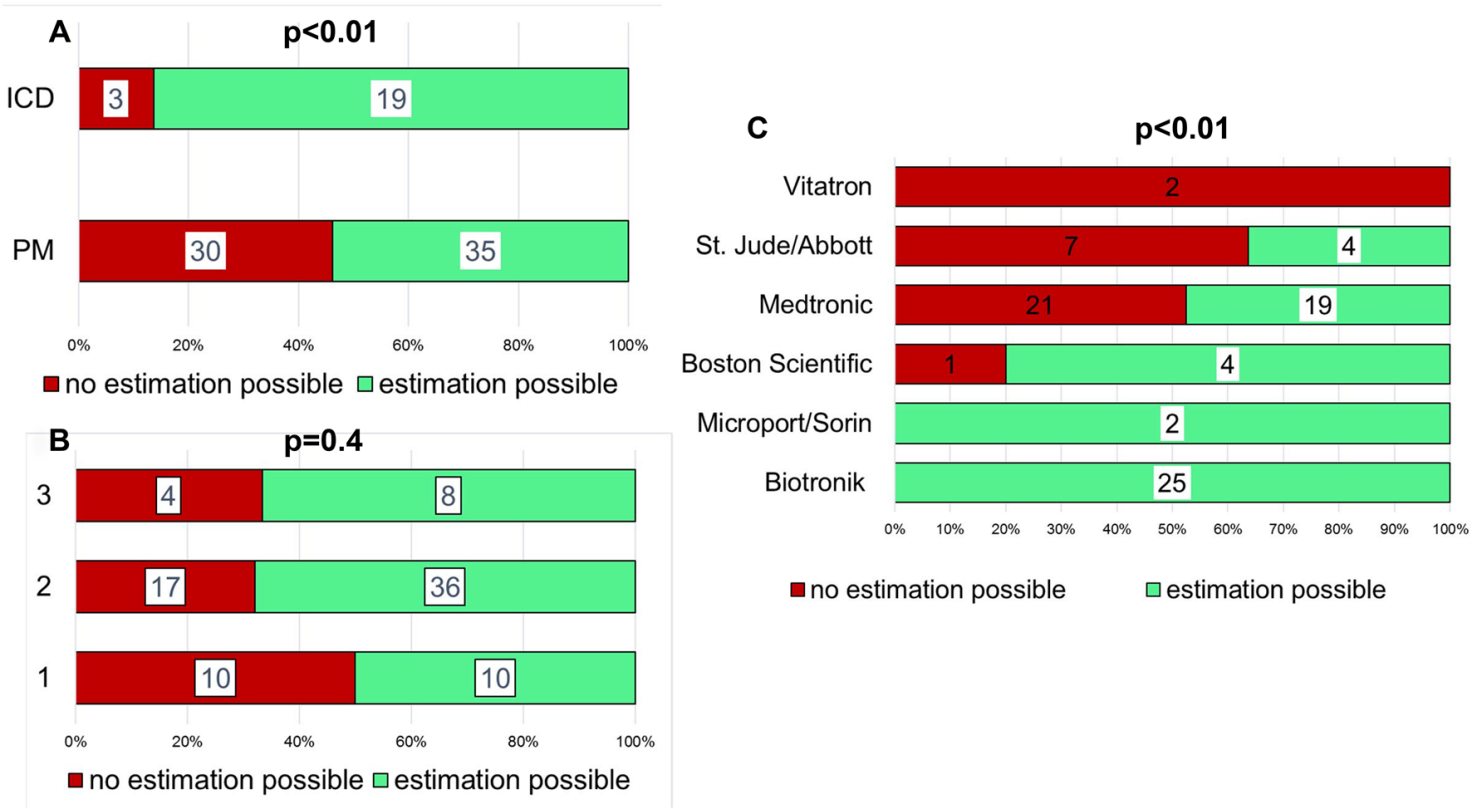
Differences in success rates of determination of time of death. (**A**): Determination of time of death was significantly more frequent in ICD devices (*p* < 0.01). (**B**): The number of leads is not significant with respect to the determination of time of death. (**C**): Relevant differences exist depending on CIED manufacturer (*p* < 0.01).

The determination of time of death was possible significantly more often in ICD (86%) compared to PM (54%; *p* < 0.01; Fig. 4A), whereas the number of leads did not significantly influence the successful determination of time of death (Fig. 4B; *p* = 0.4). Significant differences were found for the manufacturers (Fig. 4C; *p* < 0.01). The mean delay between autopsy and CIED analysis was 15 days [IQR 9-23], without a significant difference between CIED with (mean 14; IQR: 9-22) or without (mean 17; IQR: 10-25; *p* = 0.4) successful determination of time of death (supplemental Fig. 2).

### Discussion

This is the first trial to systematically evaluate whether stand-alone postmortal CIED analysis in a distant device interrogation lab can clarify the time of death in a cohort of deceased, where this remained unclear after autopsy. The major findings were:

- The overall rate of successful determination of time of death is about two thirds (62%)
- The accuracy of the determination of time of death in a blinded CIED analysis is convincing (92%)
- The determination was possible significantly more often in ICD compared with PM (86 vs. 54%; *p* < 0.01)
- Significant differences in determination rates were found between the CIED manufacturers
- Severe and potentially lethal device concerns were found in three CIED (3.4%)

The overall rate of a successful determination of time of death by CIED interrogation in the postmortal CIED trial was superior to previous reports by Lacour et al.^9^ (non-blinded interrogation, 35%) or Riesinger et al.^10^ (51%). This might have several reasons: First, the mean time between the autopsy and the CIED analysis was shorter in our trial. CIED function and storage of diagnostic data does not stop neither with patient´s death nor with removal of the CIED during autopsy. This may lead to overwriting of valuable data with each day of postmortal delay to interrogation. Second, the standardized approach for the removal of a CIED during an autopsy is performed cutting the lead^9,10^. The destruction of the leads, which remain connected to the CIED, results in artificial arrhythmia episodes, which “flood” the internal memory and may cause an overwriting of potentially lethal arrhythmia episodes. Besides, automatically conducted lead testing functions may be distorted. To overcome this issue, leads were removed by using the appropriate screw driver in the postmortal CIED trial. Third, technical development of CIED devices led to an availability of broad diagnostic data in almost any contemporary CIED which might not have been present in earlier studies.

The approach of estimation during the CIED analysis in our study was conservative and strived to refrain from any speculation. Although one primary method of estimation was listed (Fig. 3B), estimation usually based on a pattern of congruent findings. In cases of conflicting findings, no estimation was made.

The procedure was found to have a high accuracy as only one CIED in the control cohort was associated with a wrong determination of time of death. In this case, the correct time of death was missed by 1 day.

Relevant differences in the rates of successful determination were found between ICD and PM but not in correlation with the number of leads. The microcomputers in ICD are much more complex compared with PM devices and provide significantly more diagnostic information and internal memory for the storage of arrhythmia episodes. This technical difference between ICD and PM does not depend on the sole number of leads.

The rate of successful determination ranged from 0 to 100% depending on the CIED manufacturer (Fig. 4C). Even by excluding manufacturers like Microport/Sorin, Boston Scientific and Vitatron due to small numbers, relevant manufacturer specific differences persist. The determination was based on assumably lethal arrhythmia in only 24% of all successful inquires. The majority was based on the analysis of lead parameters and pacing percentages (Fig. 3B, 72%). The CIED manufactured by BIOTRONIK provide detailed information on lead parameters and percentage of pacing separated for each single day within the last 100 days. This allows a precise determination even in the absence of lethal arrhythmia.

In comparison, Medtronic CIED generate comparable diagnostic data but the information on every single day is stored only for the last 14 days. With a mean delay of 15 days between the autopsy and CIED analysis, the interrogation took place more than 14 days after death in most cases. It could be speculated that earlier analysis of Medtronic CIED might have improved success rates. Nevertheless, the delay between the autopsy and analysis of Medtronic CIED with successful determination was not significantly different from those without (15; IQR: 9–22 vs. 17; IQR 10–23; *p* = 0.8).

The assessment of device concerns in a postmortal analysis of explanted CIED is difficult, especially without any information about patient´s medical history. Previous literature on device concerns in CIED patients with sudden cardiac death by Tseng et al.^7^ designated three different kinds of device concerns: hardware concern (e.g. battery depletion), programming concern (e.g. lethal arrhythmia below the programmed therapy range in an ICD patient), selection concern (e.g. lethal arrhythmia in a patient with a PM, which could be treated by an ICD).

A hardware concern was found in three PM devices with total battery depletion. Due to protocol issues no information about operating time and regularity of CIED follow up examinations in these subjects were available. Also, the identification of programming or selection concerns was not possible within the current study, as there was no data available on potentially reasonable individual programming.

Potentially lethal arrhythmia were found in 13 CIED (7 ICD and 6 PM). It is possible, that ICD might have saved lives in the concerned PM patients. Nevertheless, it is known that lethal arrhythmia might occur even in non cardiac deaths^7^ like trauma, neurological disease or suicide. Therefore, arrhythmias may help to clarify the cause of death, but are not a proof of a cardiac arrhythmic death.

## Limitations

The interpretation of all findings is limited by the reduced number of included CIED due to the premature stop of recruitment. In particular thorough analysis comparing CIED manufacturers is impeded relevantly.

## Conclusion

Blinded postmortal analysis enables a valid determination of time of death in the majority of deceased with a CIED. The diagnostic yield was higher in ICD compared to PM devices and in BIOTRONIK devices compared to other manufacturers due to a more detailed storage of lead parameters and pacing percentages. The analysis of explanted CIED in a cardiological core lab is feasible and should be implemented in forensic autopsy.

## Supplementary Information

Supplementary Information.

## Data Availability

The data underlying this article will be shared anonymised on reasonable request to the corresponding author.

## References

[1] Moss AJ (2002). Prophylactic implantation of a defibrillator in patients with myocardial infarction and reduced ejection fraction. N. Engl. J. Med..

[2] Bardy GH (2005). Amiodarone or an implantable cardioverter-defibrillator for congestive heart failure. N. Engl. J. Med..

[3] Priori SG (2015). ESC Guidelines for the management of patients with ventricular arrhythmias and the prevention of sudden cardiac death: The task force for the management of patients with ventricular arrhythmias and the prevention of sudden cardiac death of the European society of cardiology (ESC). Endorsed by: Association for European paediatric and congenital cardiology (AEPC). Eur. Heart J..

[4] Brignole M (2013). ESC guidelines on cardiac pacing and cardiac resynchronization therapy: The Task Force on cardiac pacing and resynchronization therapy of the European Society of Cardiology (ESC). Developed in collaboration with the European Heart Rhythm Association (EHRA). Eur. Heart J..

[5] Yu CM (2005). Intrathoracic impedance monitoring in patients with heart failure: Correlation with fluid status and feasibility of early warning preceding hospitalization. Circulation.

[6] Gibson CM (2014). Design and rationale of the ANALYZE ST study: A prospective, nonrandomized, multicenter ST monitoring study to detect acute coronary syndrome events in implantable cardioverter-defibrillator patients. Am. Heart J..

[7] Tseng ZH (2015). Sudden death in patients with cardiac implantable electronic devices. JAMA Intern. Med..

[8] Sinha SK (2016). Clinical inferences of cardiovascular implantable electronic device analysis at autopsy. J. Am. Coll. Cardiol..

[9] Lacour P (2018). Cardiac implantable electronic device interrogation at forensic autopsy: An underestimated resource?. Circulation.

[10] Riesinger L (2019). Postmortem interrogation of cardiac implantable electrical devices may clarify time and cause of death. Int. J. Legal Med..

[11] Stroobandt RX, Van Heuverswyn FE, Kucher A, Barold SS (2012). Rise in ICD shock impedance: Lead fracture or death?. Pacing Clin. Electrophysiol..

[12] Duray GZ, Schmitt J, Richter S, Israel CW, Hohnloser SH (2009). Arrhythmic death in implantable cardioverter defibrillator patients: A long-term study over a 10 year implantation period. Europace.

[13] Nagele H, Hashagen S, Azizi M, Behrens S, Castel MA (2007). Analysis of terminal arrhythmias stored in the memory of pacemakers from patients dying suddenly. Europace.

[14] Pires LA, Hull ML, Nino CL, May LM, Ganji JR (1999). Sudden death in recipients of transvenous implantable cardioverter defibrillator systems: Terminal events, predictors, and potential mechanisms. J. Cardiovasc. Electrophysiol..

[15] Dyrbus M, Tajstra M, Gasior M (2019). Post mortem pro life - Should we analyse the implantable devices after death? A systematic review. Int. J. Cardiol..

[16] Basso C (2017). Guidelines for autopsy investigation of sudden cardiac death: 2017 update from the Association for European Cardiovascular Pathology. Virchows Arch..

