# Supplementary material for: Determination of time of death by blinded post-mortem interrogation of cardiac implantable electrical devices
Source: Sci Rep. 2022 May 17;12:8199. doi: 10.1038/s41598-022-12390-3 (PMC9112646; doi:10.1038/s41598-022-12390-3)
Supplement: Supplementary file 1 — Supplementary Information. [file 41598_2022_12390_MOESM1_ESM.pptx]

## Slide 1
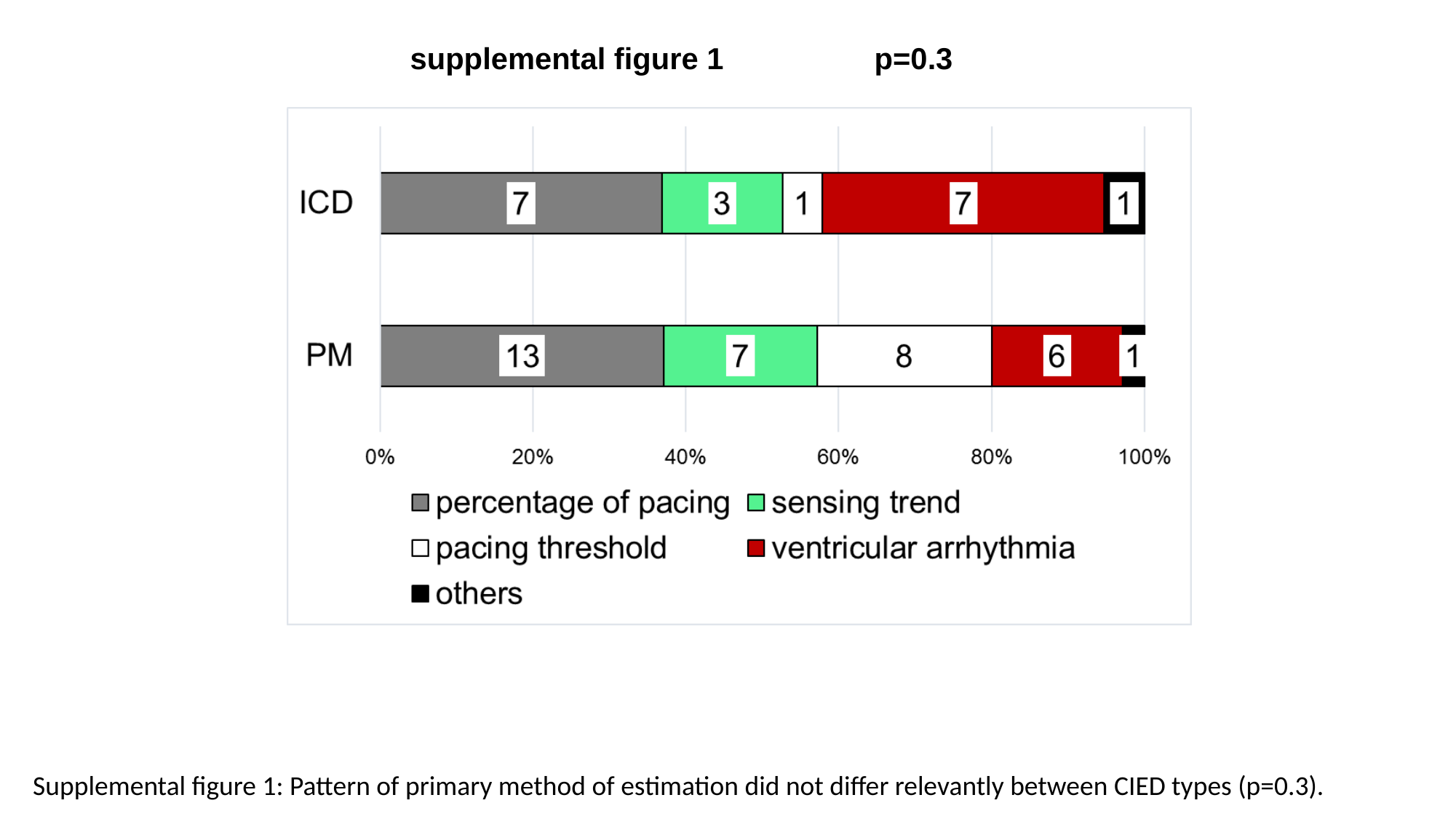

supplemental figure 1 p=0.3
Supplemental figure 1: Pattern of primary method of estimation did not differ relevantly between CIED types (p=0.3).

## Slide 2
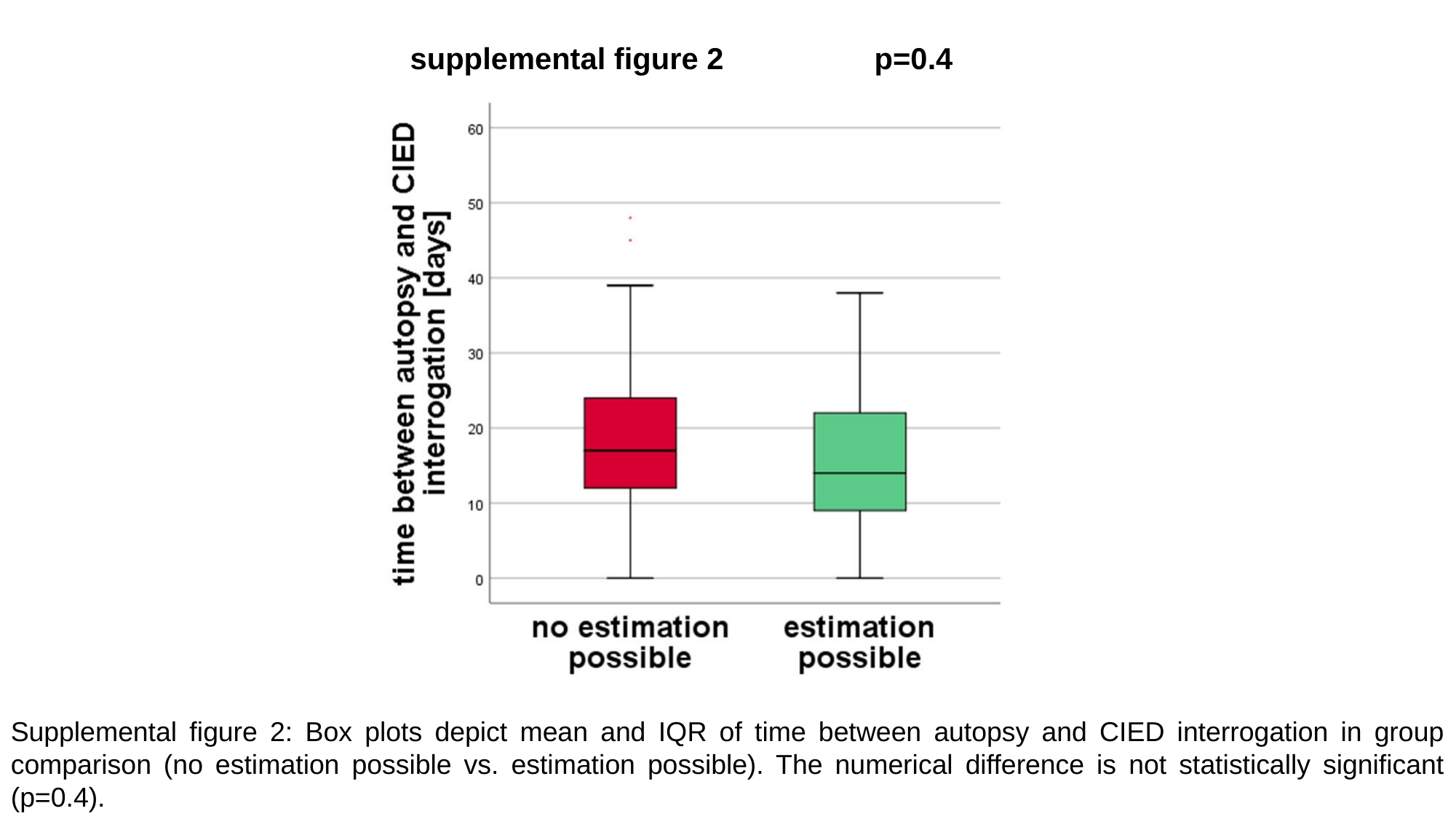

supplemental figure 2 p=0.4
Supplemental figure 2: Box plots depict mean and IQR of time between autopsy and CIED interrogation in group comparison (no estimation possible vs. estimation possible). The numerical difference is not statistically significant (p=0.4).

## Slide 3
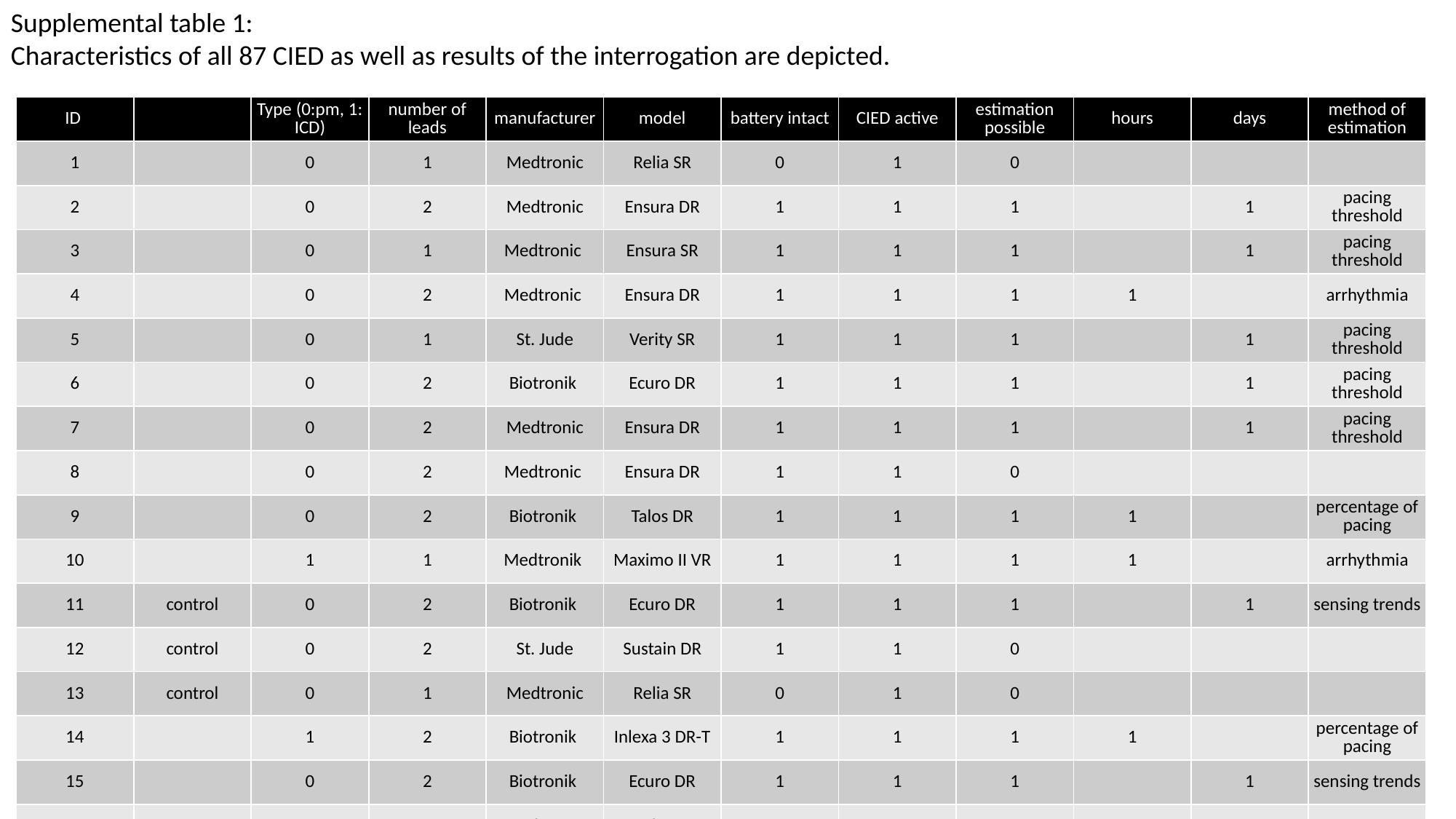

Supplemental table 1:
Characteristics of all 87 CIED as well as results of the interrogation are depicted.
| ID | | Type (0:pm, 1: ICD) | number of leads | manufacturer | model | battery intact | CIED active | estimation possible | hours | days | method of estimation |
| --- | --- | --- | --- | --- | --- | --- | --- | --- | --- | --- | --- |
| 1 | | 0 | 1 | Medtronic | Relia SR | 0 | 1 | 0 | | | |
| 2 | | 0 | 2 | Medtronic | Ensura DR | 1 | 1 | 1 | | 1 | pacing threshold |
| 3 | | 0 | 1 | Medtronic | Ensura SR | 1 | 1 | 1 | | 1 | pacing threshold |
| 4 | | 0 | 2 | Medtronic | Ensura DR | 1 | 1 | 1 | 1 | | arrhythmia |
| 5 | | 0 | 1 | St. Jude | Verity SR | 1 | 1 | 1 | | 1 | pacing threshold |
| 6 | | 0 | 2 | Biotronik | Ecuro DR | 1 | 1 | 1 | | 1 | pacing threshold |
| 7 | | 0 | 2 | Medtronic | Ensura DR | 1 | 1 | 1 | | 1 | pacing threshold |
| 8 | | 0 | 2 | Medtronic | Ensura DR | 1 | 1 | 0 | | | |
| 9 | | 0 | 2 | Biotronik | Talos DR | 1 | 1 | 1 | 1 | | percentage of pacing |
| 10 | | 1 | 1 | Medtronik | Maximo II VR | 1 | 1 | 1 | 1 | | arrhythmia |
| 11 | control | 0 | 2 | Biotronik | Ecuro DR | 1 | 1 | 1 | | 1 | sensing trends |
| 12 | control | 0 | 2 | St. Jude | Sustain DR | 1 | 1 | 0 | | | |
| 13 | control | 0 | 1 | Medtronic | Relia SR | 0 | 1 | 0 | | | |
| 14 | | 1 | 2 | Biotronik | Inlexa 3 DR-T | 1 | 1 | 1 | 1 | | percentage of pacing |
| 15 | | 0 | 2 | Biotronik | Ecuro DR | 1 | 1 | 1 | | 1 | sensing trends |
| 16 | | 0 | | unkown | unkown | 0 | 0 | 0 | | | |
| 17 | control | 0 | 2 | Medtronic | Sensia DR | 1 | 1 | 1 | | 1 | pacing threshold |
| 18 | control | 1 | 2 | Biotronik | Lumax DR-T | 1 | 1 | 1 | | 1 | percentage of pacing |
| 19 | | 1 | 3 | Medtronic | Protecta CRT-D | 1 | 1 | 0 | | | |
| 20 | control | 0 | 2 | Biotronik | Enticos DR | 1 | 1 | 1 | | 1 | percentage of pacing |
| 21 | control | 0 | 2 | Biotronik | Talos DR | 0 | 1 | 1 | 1 | | percentage of pacing |
| 22 | | 0 | 2 | Medtronic | Relia DR | 0 | 1 | 0 | | | |
| 23 | control | 0 | 3 | Biotronik | Enitra 8 HF | 1 | 1 | 1 | | 1 | percentage of pacing |
| 24 | | 0 | 2 | Biotronik | Ecuro DR | 1 | 1 | 1 | 1 | | arrhythmia |
| 25 | | 0 | 2 | St. Jude | Endurity 2K | 1 | 1 | 1 | | 1 | additional diagnostics |
| 26 | control | 1 | 3 | Medtronic | Brava Quad | 1 | 1 | 1 | 1 | | arrhythmia |
| 27 | control | 0 | 1 | Medtronic | Ensura SR | 1 | 1 | 1 | | 1 | sensing trends |
| 28 | control | 1 | 1 | Biotronik | Rivacor VR-T | 1 | 1 | 1 | | 1 | pacing threshold |
| 29 | | 1 | 1 | Medtronic | Protecta VR | 0 | 1 | 1 | 1 | | arrhythmia |
| 30 | | 0 | 1 | St. Jude | Verity | 0 | 0 | 0 | | | |
| 31 | control | 0 | 1 | Biotronik | Effecta SR | 1 | 1 | 1 | | 1 | sensing trends |
| 32 | control | 0 | 2 | Medtronic | Sensia DR | 1 | 1 | 1 | 1 | | arrhythmia |
| 33 | control | 0 | 2 | St. Jude | Endurity Core | 1 | 1 | 0 | | | |
| 34 | | 0 | 2 | Biotronik | Enitra 8 DR-T | 1 | 1 | 1 | | 1 | sensing trends |
| 35 | | 1 | 1 | Medtronic | Protecta VR | 1 | 1 | 0 | | | |
| 36 | | 0 | 2 | Vitatron | T70 DR | 0 | 1 | 0 | | | |
| 37 | control | 0 | 2 | St. Jude | Sustain DR | 1 | 1 | 0 | | | |
| 38 | control | 0 | 2 | Medtronic | Relia DR | 0 | 1 | 0 | | | |
| 39 | | 0 | 2 | Boston Scientific | Essentio DR | 1 | 1 | 1 | 1 | | arrhythmia |
| 40 | | 0 | 2 | Medtronic | Sensia DR | 1 | 1 | 0 | | | |
| 41 | | 0 | 1 | Medtronic | Sensia VR | 0 | 1 | 0 | | | |
| 42 | control | 1 | 3 | Boston | Inogen CRT-D | 1 | 1 | 1 | 1 | | arrhythmia |
| 43 | | 0 | 2 | Medtronic | Ensura DR | 1 | 1 | 1 | 1 | | arrhythmia |
| 44 | | 0 | 3 | Biotronik | Entovis HF-T | 1 | 1 | 1 | | 1 | sensing trends |
| 45 | | 1 | 2 | Microport | Paradym 2 DR | 1 | 1 | 1 | 1 | | percentage of pacing |
| 46 | | 1 | 2 | St. Jude | Ellipse DR | 1 | 1 | 1 | | 1 | sensing trends |
| 47 | | 0 | 1 | Vitatron | T20 SR | 0 | 1 | 0 | | | |
| 48 | | 1 | 1 | Biotronik | Iforia 3 VR-T | 1 | 1 | 1 | | 1 | sensing trends |
| 49 | | 0 | 2 | Biotronik | Ecuro DR | 1 | 1 | 1 | | 1 | percentage of pacing |
| 50 | | 0 | 2 | Medtronic | Ensura DR | 1 | 1 | 0 | | | |
| 51 | | 1 | 1 | Medtronic | Mirro VR | 1 | 1 | 1 | 1 | | arrhythmia |
| 52 | | 0 | 2 | St. Jude | Sustain DR | 1 | 1 | 0 | | | |
| 53 | | 0 | 2 | Medtronic | Astra XT DR | 1 | 1 | 1 | | 1 | pacing threshold |
| 54 | control | 0 | 2 | Medtronic | Ensura DR | 1 | 1 | 1 | | 1 | pacing threshold |
| 55 | | 1 | 3 | Boston Scientific | Inogen CRT-D | 1 | 0 | 1 | 1 | | arrhythmia |
| 56 | | 0 | 1 | Medtronic | Relia SR | 0 | 1 | 0 | | | |
| 57 | control | 0 | 2 | St. Jude | Sustain DR | 1 | 1 | 0 | | | |
| 58 | | 0 | 2 | Biotronik | Enticos 4 DR | 1 | 1 | 1 | | 1 | percentage of pacing |
| 59 | | 0 | 2 | Biotronik | Entovis DR-T | 1 | 1 | 1 | | 1 | sensing trends |
| 60 | | 0 | 3 | Medtronic | Consulta CRT-P | 1 | 1 | 0 | | | |
| 61 | | 0 | 2 | Biotronik | Effecta DR | 1 | 1 | 1 | 1 | | percentage of pacing |
| 62 | | 0 | 2 | Biotronik | Effecta DR | 1 | 1 | 1 | 1 | | percentage of pacing |
| 63 | | 0 | 2 | Biotronik | Evia DR-T | 1 | 1 | 1 | 1 | | percentage of pacing |
| 64 | | 0 | 2 | Medtronic | Ensura DR | 1 | 1 | 0 | | | |
| 65 | | 0 | 1 | Medtronic | Relia SR | 0 | 1 | 0 | | | |
| 66 | | 1 | 3 | Medtronic | Amplia Quad CRT-D | 1 | 1 | 1 | 1 | | arrhythmia |
| 67 | | 1 | 2 | Medtronic | Mirro DR | 1 | 0 | 1 | | 1 | percentage of pacing |
| 68 | control | 0 | 3 | Medtronic | Serena Quad CRT-P | 1 | 1 | 0 | | | |
| 69 | | 0 | 2 | Biotronik | Ecuro DR | 1 | 1 | 1 | 1 | | percentage of pacing |
| 70 | | 0 | 1 | Medtronic | Sensia SR | 1 | 1 | 0 | | | |
| 71 | | 1 | 1 | Biotronik | Iforia 5 VR-T | 1 | 1 | 1 | 1 | | percentage of pacing |
| 72 | | 0 | 2 | Medtronic | Ensura DR MRI | 1 | 1 | 1 | 1 | | arrhythmia |
| 73 | | 0 | 2 | Microport | Teo DR | 1 | 1 | 1 | 1 | | percentage of pacing |
| 74 | | 1 | 2 | St. Jude | Ellipse DR | 1 | 1 | 1 | | 1 | additional diagnostics |
| 75 | | 0 | 2 | St. Jude | Integrity DR | 0 | 1 | 0 | | | |
| 76 | control | 0 | 2 | Medtronic | Adapta DR | 0 | 1 | 0 | | | |
| 77 | | 1 | 3 | Boston Scientific | Charisma X4 CRT-D | 1 | 1 | 0 | | | |
| 78 | | 0 | 1 | Medtronic | Relia SR | 0 | 1 | 0 | | | |
| 79 | | 1 | 3 | Medtronic | Viva XT CRT-D | 0 | 1 | 1 | | 1 | percentage of pacing |
| 80 | | 0 | 2 | Biotronik | Ecuro DR | 1 | 1 | 1 | 1 | | percentage of pacing |
| 81 | | 1 | 2 | Biotronik | Ecuro DR | 1 | 1 | 1 | 1 | | percentage of pacing |
| 82 | | 0 | 2 | Medtronic | Attesta DR | 1 | 1 | 0 | | | |
| 83 | | 0 | 3 | Medtronic | Solara CRT-P | 1 | 1 | 1 | | 1 | percentage of pacing |
| 84 | | 1 | 2 | Boston Scientific | Inogen ICD 2K | 1 | 1 | 1 | | 1 | sensing trends |
| 85 | | 0 | 2 | Medtronic | Sensia DR | 0 | 1 | 0 | | | |
| 86 | | 0 | 2 | Medtronic | Sensia DR | 1 | 1 | 0 | | | |
| 87 | | 0 | | unknown | unknown | 0 | 0 | 0 | | | |
